# C-753-05. Peripherally Inserted Central Line Catheters Are Safe to Use in Burn Patients

**DOI:** 10.1093/jbcr/irag033.026

**Published:** 2026-04-06

**Authors:** Theophilus Pham, Alura L Barsun, Andrew Fish, Miyoung So, Jason Heard, Soman Sen, Tina L Palmieri, Kathleen S Romanowski

**Affiliations:** Firefighters Burn Institute Regional Burn Center - University of California Davis, Sacramento, CA; University of California Davis Health, Sacramento, CA; University of California Davis Health, Sacramento, CA; University of California Davis Health, Sacramento, CA; Firefighters Burn Institute Regional Burn Center - University of California Davis, Sacramento, CA; University of California Davis Health, Sacramento, CA; University of California Davis Health, Shriners Children’s - Northern California, Sacramento, CA; University of California Davis Burn Center & Shriners Hospital for Children, Sacramento, CA

## Abstract

**Introduction:**

Peripherally inserted central catheters (PICCs) are increasingly used in burn care as an intermediate option between peripheral intravenous lines and central venous catheters. While central line–associated bloodstream infections (CLABSIs) in burn patients are well characterized, the specific risks associated with PICCs in this population remain poorly defined. Improved quantification of complication rates, identification of risk factors, and evidence-based protocols for PICC management are needed in burn care.

**Methods:**

We performed a retrospective chart review of all burn patients at our ABA-verified regional burn center having a PICC order placed between January 2020 and December 2024. Data collected included demographics, injury characteristics, PICC related variables, and infection related information. We calculated infection rates per 1000 PICC days and performed both univariate and multivariate analyses to identify risk factors associated with PICC infection. Continuous variables are reported as median (interquartile range). Statistical significance was defined as *p*<.05.

**Results:**

We identified 436 unique patients accounting for 525 PICC orders; of these, 419 PICCs were ultimately placed. Reasons for nonplacement included positive blood cultures (21, 4%), patient refusal (20, 3.8%), and presence of burn injury at insertion site (20,3.8%). Indications for PICC use included need for multilumen access (189, 36%), need for vasoactive medication (14, 2.7%), and inability to maintain peripheral IV access (129, 24.6%). The cumulative PICC infection rate was 4.7 infections per 1000 PICC-days. Patients who developed PICC infections had higher mean TBSA (30% vs 17.5%, *p*=.0006) and longer hospital stays (67 days vs 29 days, *p*<.0001). On univariate analysis, infected patients also had longer intervals from admission to PICC placement (19.5 days vs 8 days, *p*=.0002) and a higher prevalence of inhalation injury (43.3% vs 17.5%, *p*=.0006). There was no statistically significant increase in infection risk associated with burn injury of the upper extremity or burns on the same limb used for PICC insertion. In multivariate logistic regression, inhalation injury (odds ratio [OR] = 2.9) and placement for vasoactive medications (OR = 5.2) emerged as independent predictors of PICC infection.

**Conclusions:**

In burn patients, PICC-associated infection rates appear lower than historically anticipated. However, certain subgroups, particularly those with inhalation injury and vasoactive medication requirements, are at markedly increased risk. These findings support the need for a risk stratification or scoring system to guide early PICC insertion in suitable candidates, close monitoring while in situ, and prompt removal when no longer needed.

**Applicability of Research to Practice:**

PICC lines can safely be used in burn patients with care taken for patient selection and removal when the line is no longer required.

**Funding for the Study:**

N/A.

